# Modelling the effects of climate change on shellfish production in marine artisanal fisheries of Ghana

**DOI:** 10.12688/aasopenres.12956.2

**Published:** 2025-01-16

**Authors:** Sandra Akugpoka Atindana, Patrick Kwabena Ofori-Danson, Sandra Brucet

**Affiliations:** 1 Department of Fisheries and Aquatic Resources Management, University for Development Studies, Tamale, Ghana; 2 Department of Fisheries and Aquaculture, School of Agriculture, CK Tedam University of Technology and Applied Sciences, Navrongo, Ghana; 3 Department of Marine and Fisheries Sciences, University of Ghana, Accra, Ghana; 4 Aquatic Ecology Group, University of Vic – Central University of Catalonia, Spain. ICREA and University of Vic - Central University of Catalonia. . U Science Tech (UST)., Vic, Spain; 5 ICREA, Catalan Inst. for Research and Advanced Studies, Barcelona, Spain

**Keywords:** Climate change, Model, Shellfish catch, Oyster, Prediction, Production

## Abstract

**Background:**

Ghana’s marine artisanal fisheries, particularly the small pelagic fisheries, are in a state of crisis. The decline in the number of small pelagic fish are attributable to overfishing, climate variability and unsustainable fishing methods. Similarly, in the wake of climate change, shellfishes (particularly oysters, scallops and mussels) are highly vulnerable.

**Methods:**

A total of 55 years’ worth of data from Ghana’s marine artisanal fisheries were studied in relation to climate indices. The primary objective was to develop a simple linear regression model for predicting shellfish catch in Ghana. Key informant interviews were employed in soliciting data on changes in climate along the coastline and trends in marine artisanal shell fish catch.

**Results:**

The predictor variable that significantly explained shellfish production was temperature. Hence, the model is a valuable tool to predict future trends in the shellfish catch in marine artisanal fisheries.

**Conclusions:**

Increases in sea surface temperature will adversely affect shellfish production. It is therefore important that the Ministry of Fisheries and Aquaculture Development and other stakeholders should, in their decision-making processes, ensure the formulation of climate smart policies and management strategies for sustainable use of the resource.

## Introduction

Ghana has a coastline of approximately 550 km and a continental shelf area of 24,300 km ( Asare *et al.*, 2015). The country has the fifth largest exclusive economic zone (EEZ) in West Africa and is known to have the most vibrant fisheries in Africa. The average annual per capita consumption of fin and shell fish is 27.3 kg, which accounts for 60% of animal protein consumption ( USAID-BC, 2016). The coastline of Ghana also supports diverse aquatic ecosystems.

Adjacent to the coast are numerous brackish waters. These systems mainly estuaries and lagoons drain into the Gulf of Guinea and form critical habitats for many marine animals, such as shellfish, finfish and other organisms such as migratory birds. Brackish waters serve as sinks for flood control, stabilise shorelines and mitigate climate as a result of the presence of mangrove ecosysems. Any adverse effects of climate and human induced stressors on the coastline of Ghana will impact largely on all interdependent ecosystems. Furthermore, marine artisanal fisheries of Ghana is of staunched importance to the country through the provision of fish particularly the small pelagic fishes, livelihood sources and food. There are well-documented data on the fish of commercial importance; however, these official data fail to include the catches of non-commercial sectors, especially shellfisheries ( Pauly *et al.*, 2002). Despite this, the artisanal sector primarily dominates total catch, while the industrial sector contributes secondarily to total catch. The importance of non-commercial sectors, for example those surrounding bivalves and crustaceans, in eliminating food insecurity and malnutrition, cannot be relegated to the background.

Shellfish, such as oysters, scallops, mussels and crustaceans, are a main source of food and income for small-scale fishers in the coast of Ghana ( Obodai & Yankson, 1999). Ecologically, some groups of shellfish, such as the West African oyster ( *Crassostrea tulipa)* and scallops, are used in the biomonitoring of pollution as they have the capacity to filter pollutants, and the mangrove ecosystems in which they thrive absorb 5–8 times of atmospheric carbon than terrestrial ecosystems thus reducing global warming and mitigating the effects of climate change in coastal aquatic ecosystems ( NOAA, 2013). The National Oceanic and Atmospheric Administration ( NOAA, 2013) reported the use of both oysters and mussels to monitor concentrations of trace metals in water bodies throughout the world. Hence a stable oyster production is an indication of good water quality. In relation to human nutrition, oysters are lean, low-calorie, high-quality protein that contain omega-3 fatty acids and are low in saturated fat. Oysters and scallops are used as food, providing protein and minerals to humans. The shells of oysters are used to prepare feed for birds, medicine for humans and are ingredients in paint ( Ansa & Bashir, 2007; Obodai & Yankson, 1999). Similarly, mussels are a good source of omega 3 fatty acids for human nutrition.

Global production of marine capture fisheries is decreasing ( FAO, 2018). Catches declined from 92.2 million tonnes (MT) in 2014 to 90.9 MT in 2016 ( FAO, 2018). Similarly, production from marine artisanal capture fisheries has decreased to 79.3 MT from as high as 79.9 MT in the year 2014 ( FAO, 2018). Despite the decline in catches, the average Ghanaian relies on fish for about 60% of the animal protein consumed ( USAID-BC, 2016). The causes of these declines are attributable to natural and anthropogenic factors. Unsustainable fishing methods and overfishing may likely increase the effects of natural drivers such as climate change. Variability in climate has become an issue of global concern. Global trends in temperature reflect a rise of 0.85–1.06°C, while sea level rise is about 3.2 mm per year between 1993 and 2010. A continual increase in temperature, rainfall, intensity of floods and decrease in rainfall renders Ghana’s coastal shellfisheries highly vulnerable.

The impact will stem from resultant effects of increasing temperature and ocean acidification on their physiological, morphological, reproductive, migratory and behavioural responses. Ghana experiences a biannual upwelling season, which brings cold water and large quantity of fish to its shore, resulting in fish abundance and subsequent reduction in fish catch after the phenomenon. Human factors further exacerbate the incidences of low fish catch. Among such factors are overfishing, unauthorised fishing methods and low enforcement of regulations. Whereas anthropogenic stressors may be regulated, natural factors are complex to deal with. The variability in production from natural drivers requires efforts to be put in place to address climate change in the fisheries sector. Among some of these efforts are the development of models for predicting the impact of climate change on fisheries and the adoption of sustainable adaptation strategies for increased resilience and sustainable fisheries.

The formulation of management strategies for sustainable conservation of aquatic resources require the adoption of useful predictive tools. Among these tools are regression models, which are excellent for projecting future changes in natural aquatic systems ( Rocha *et al.*, 2009). Conversely, despite the fact that future predictions using regression models involving a small number of variables with large data sets are essential, very few of these models have been developed for tropical coastal systems and fisheries ( Pace, 2001; Rocha *et al.*, 2009). In the fourth assessment report of the first working group at a symposium organised by the Intergovernmental Panel on Climate Change (IPCC AR4), a joint study by Intergovernmental Council for the Exploration of the Sea (ICES) and the North Pacific Marine Science Organisation (PICES), indicates several recent interdisciplinary attempts in deepening our knowledge on the effects of climate forcing on aquatic life by enhancing the development of predictions on climate variability effects on various stages of fish growth ( Brander, 2008; Hollowed *et al.*, 2008; ICES, 2008).

As part of contributing knowledge to this debate, the main goal of this research is to generate relevant data on climate variability effects on shellfish in the country’s coastal plain and its implication on some vulnerable groups of shellfish for the formulation of policies and management plans in the wake of climate change. The study aimed at addressing two research questions; (i) what is the trend in shellfish production in the marine artisanal fisheries over the years? And (ii) how will climate indices influence shellfish catch particularly the vulnerable groups (oysters, scallops and mussels) in the marine artisanal sector of Ghana?

### Marine fish production in Ghana over the years

There have been increases in Ghanaian domestic marine fisheries catch in the early 1970’s from as low as 63,000 tonnes in 1950 to 415,000 tonnes ( FAO, 2016). The reason for the increase in marine catches, according to FAO reports, was due to the increase in the number of fishers from around 7,400 tonnes/year in 1950 to 21,800 tonnes/year in 2010 MOFAD (2015). This concurrently necessitated an increase in fish export to sustain per capital fish consumption in the world, with Ghana being no exception ( FAO, 2016; FAO, 2018; Nunoo *et al.*, 2014). During the coup in 1972, Ghana’s marine fish production level recorded a sharp decrease to about 260,000 tonnes until the late 1990s where catch gradually increased to approximately 440,000 tonnes ( Nunoo *et al.*, 2014). Thereafter, marine fisheries production and export has been declining with decreases ranging between 420,000 tonnes in 1999 to 202,000 tonnes in 2014 ( FAO, 2018). Fish export rate decreased from US Dollar (USD) 120 million in 2003 to USD 44 million in 2014.

### Climate change and food security

The impact of climate change on human and natural ecosystems is of recent concern. Prominent among these impacts are the detrimental effects of climate change on the environment and global food security. Future projections reveal that global food production systems will be affected by many factors, such as drought and floods due to changes in temperature and in precipitation patterns ( IPCC, 2007). These changes will also affect the entire food value chain particularly food marketing systems and directly affect food affordability ( Brander, 2007; Gregory *et al.*, 2005). These effects are noted to affect both terrestrial and aquatic systems. Among aquatic ecosystems, marine ecosystems which are closely tied to the sea are envisaged to be most vulnerable. Tremendous efforts have been put in place in an attempt to addressing the impacts of climate change on agriculture. Meanwhile, climate change also poses threats to the sustainability of the capture fishery and aquaculture sector mainly in the form of food insecurity and livelihoods of millions of people ( Cochrane *et al.*, 2009; FAO, 2008; WFC, 2009). Furthermore, most fishery dependent communities are located in countries highly exposed to climate change. Among these countries are Western and Sub-Saharan Africa, north-western South America and Asia ( Allison *et al.*, 2009; WFC, 2009).

### Effects of climate change on shellfishes

In Sub-Saharan Africa shellfish will be more badly hit by climate change in comparison with finned fish. Increased greenhouse gas emissions will have direct and indirect effects on bivalves and gastropods. The resultant effects of climate change are of potential impact on the habitat and the organisms. Global warming, sea level rise and ocean acidification may lead to alteration of the habitats supporting invertebrate life forms. Channel banks, sand and mud flats are habitats suitable for shellfish. Climate change will affect these habitats with resultant effects from tidal inundation and tidal velocities which may likely degrade these areas and result in decline in fish abundance.

Additionally, shellfish abundance may decline due to increasing temperature, lowering pH, light and hydrology affecting physiological performance and fitness ( Barton *et al.*, 2012; Johnson *et al.*, 2018). Ocean acidification will inhibit the ability of shellfish and other shell-bearing organisms from building their protective coverings made of calcium carbonate ( Barton *et al.*, 2012; La Peyre *et al.*, 2009 as cited in Atindana *et al.*, 2019). The ability of calcifying plankton to produce food for herbivorous shellfish will be affected. For example, the formation of frustules of diatoms may be challenged in acidified waters. Additionally, increases in ocean acidification are lethal to shellfish growth. Fish recruitment will be affected due to reduced sensory responses, predator avoidance and individual behaviour. Another climate factor that is a threat to fish survival is sea surface temperature (SST). Increasing SST is predicted to have immediate and greater impact on invertebrates and demersal fishes despite their adaptation to varying diurnal and seasonal temperature cycles. Increasing SST will affect the ability of fish and shellfish to reproduce successfully, recruit stocks, grow and survive ( Donelson *et al.*, 2010; Pratchett *et al.*, 2008).

### Fishing efforts in marine artisanal shell fisheries of Ghana from 1970 – 2024

In the 1970s–1990s there was low-to-moderate fishing pressure in the marine artisanal sector of the country. Fishing efforts were primarily limited by the availability of simple, non-motorized equipment and the reliance on traditional ecological knowledge ( MOFAD, 2019). Traditional practices such as seasonal closures ("nnabone") regulated fishing effort to some extent. These culturally embedded restrictions aligned with ecological cycles, such as spawning seasons. Population growth and rising coastal communities led to increased reliance on marine resources. Artisanal shellfish fisheries targeted species like oysters, scallops, and clams, primarily for local consumption ( Adom *et al.*, 2019; Watson & Pauly, 1993).

From the 2000s there have been increase use of technology thus the adoption of motorized canoes and improved fishing gear (e.g., fine-mesh nets) which enabled higher catch efficiency but also led to overharvesting of shellfish resources. This has led to the expansion of fishing grounds including nearshore and estuarine areas, further stressing habitats. The open-access fishing regimes led to uncontrolled fishing effort, contributing to the depletion of shellfish stocks and a shift from subsistence to commercial fishing increased the intensity of resource extraction. Overfishing, combined with environmental degradation (e.g., mangrove deforestation), led to declines in shellfish stocks, reducing catch sizes and impacting fisher livelihoods ( IOI, 2020; MOFAD, 2019).

From the 2010s–2024, policies like closed seasons were introduced (e.g., May–June seasonal bans) to reduce fishing pressure and allow stock recovery. Despite that enforcement of regulations remains a challenge due to limited resources and resistance from local communities, who depend on fisheries for livelihoods ( FAO, 2024; MOFAD, 2019).

Meanwhile, rising sea temperatures and habitat destruction have further reduced shellfish availability, intensifying competition among fishers ( Boateng, 1998; Yidan *et al.*, 2024). Degradation of spawning and nursery habitats, such as mangroves, exacerbates the decline in resource productivity. Sociocultural and economic dynamics can not be underestimated. Economic pressures have driven fishers to increase effort, sometimes using illegal or destructive methods (e.g., chemicals, small-mesh nets). Artisanal fisheries continue to struggle with resource conflicts, including competition with industrial trawlers encroaching on artisanal zones. 

Conclusively, the trends in fishing effort in the sector have metamorphosised from modest, subsistence-oriented efforts in the 1970s to an incremental rise in fishing intensity driven by technological advances, population growth, and commercialization.

### Historical trends on fishing pressure from 1970s–1990s

In the early years of the 70s and 90s the marine artisanal fisheries grew rapidly, serving as a critical livelihood and food source. During this period, open access to fishing resources led to increased effort, including the adoption of unsustainable practices like using small-mesh nets. Also, cultural practices such as seasonal fishing closures (e.g., "nnabone") were observed, coinciding with breeding seasons and helping to sustain fish and shellfish stocks ( MOFAD, 2019).

### Historical trends on fishing pressure from 2000s–2020s

Population growth and rising demand for seafood intensified fishing activities, including overharvesting shellfish such as oysters and clams. Illegal, unreported, and unregulated (IUU) fishing further exacerbated stock depletion ( IOI, 2020; Watson & Pauly, 1993).

These challenges is heightened by weak enforcement of fisheries regulations allowing harmful practices to persist, despite the introduction of policies like closed seasons (e.g., May–June closures in recent years). Some communities resisted closures, citing economic hardships during no-fishing periods ( Adom *et al.*, 2019).

In adapting to this, artisanal fishers have shifted towards alternative methods and targeted other species due to the declining availability of traditional shellfish resources.

Fisheries management institutions and experts in the country have come up with some developments and efforts. These efforts stem from alternative livelihoods to reduce pressure on shellfisheries while supporting fisher communities' resilience and reviving seasonal closures and advocacy programmes by researchers for the integration of traditional and modern management systems. Seasonal closures have met mixed success, with improved yields in some areas but limited compliance in others. The assumption in this study is that fishing pressure is held constant throughout the years.

## Methods

### Study area

**Figure 1.**
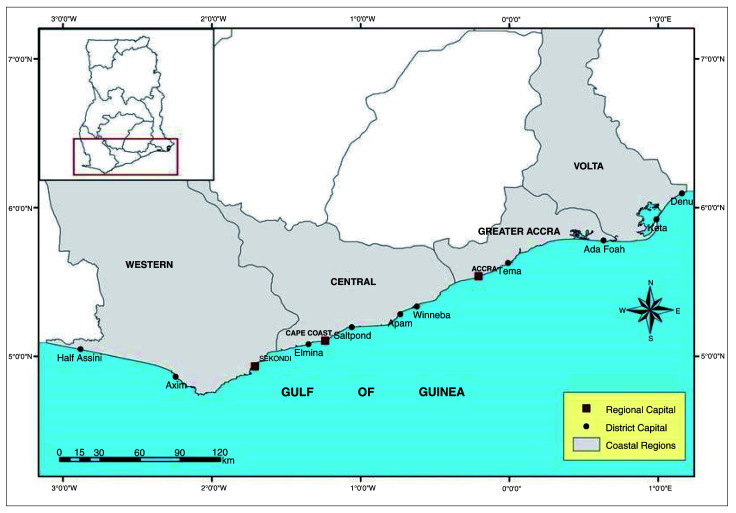
Map of the coastline of Ghana showing Ghana’s coastal regions.

The research was conducted across the coastal plains of Ghana in the Volta region, Greater Accra, Western and Central regions of Ghana; Keta, Tema, Takoradi and Elmina fishing harbours respectively ( Figure 1). This was achieved by collating catch data from the four fishing areas obtained from the Fisheries Scientific Survey Division (FSSD) at Tema, MOFAD Ghana.

Tema metropolis is located 30 kilometres East of Accra, Ghana. Northeast of Tema is the Dangbe West District. It is bordered by Ledzokuku Krowor Municipality to the southwest and Adentan and Ga East Municipality to the northwest. Northeast, and South of Tema are Akuapim South District and the Gulf of Guinea, respectively. Tema has an area of about 87.8 km ^2^ and a generally flat land surface. The terrain of Tema barely rises up to 35 m above sea level ( GSS, 2014). South East of Tema, lies the Keta coastline in Volta Region.

The Keta coastline is located 160 km from Accra and lies within Longitude 0.30° E and 1.05° E and Latitude 5.45° N and 6.005° N. North of Keta is the Akatsi South District. The Ketu North and South District are located east of Keta. Keta is a low-lying coastal plain. The area is prone to coastal erosion ( GSS, 2014). Southwest of Keta lies the Takoradi coastal fishing area.

 Takoradi is located approximately 210 km west of Accra It shares boundaries with Mpohor Wassa East district to the north, Shama District to the east, Ahanta West district to the west and the Gulf of Guinea to the south. Takoradi lies 6 m below sea level (STMA, forthcoming). The main features of the Takoradi coastline is its predominating sandy beaches, rocky headlands, near shore rocky bottoms and engineered structures. There are incidences of coastal erosion and in the past few decades, the area has eroded not less than 10 to 100 m ( CRC/FON, 2010; GSS, 2014).

### Sampling design

***Collection of historic data on shellfish production.*** Owing to a lack of documented data on oyster catch in Ghana, historic marine artisanal shellfish catch data from 1970–2015 from the Ministry of Fisheries and Aquaculture Development ( MOFAD, 2016); Tema and FAO (2016) were collected and used to draw scenarios for possible effects on vulnerable groups of shellfish in Ghana. The shellfish groups comprised bivalves, gastropods and shrimps, and excluded cephalopods. Incomplete annual catch data were filled by first calculating an average value from pre-existing data (2013–2015). This value was an estimation of the percentage contribution of shellfish to the overall marine capture fisheries catch, as reported in the FAO Fisheries Statistics ( FAO, 2016). Annual shellfish production data from 1970 to 2000 was estimated using the formula below:

$\text{Shellfish}\;\text{production}=\frac{\text{Average}\;\text{value}}{100}\times \text{Overall}\;\text{marine}\;\text{catch}\;\text{in}\;\text{each}\;\text{year}$

In filling in data, the difference in catch for the year duration was taken and divided by the number of gaps in the years and then subsequently added or subtracted by previous or preceding years, depending on the trend in catch. An extensive literature review was carried out to obtain data related to shellfish production. FAO reports and MOFAD records on available shellfish groups were collated to obtain approximations of yearly catch from 1970 to 2015.

Personal interviews with two experts from the MOFAD and GMet were employed to gather information on catch and climate in the country. No written ethical approval was sought because of the low-risk nature of the conversations. Through personal communication, the experts shared their working and research experiences on shellfish fishery and weather patterns in Tema without restraints. Questions from the interview concerned the number of shellfish groups in the marine artisanal fisheries (shrimps, bivalves), harvesting methods, trends in shellfish catch, calculation of missing data using the moving average method, limitations in the fishery, complex weather patterns such as El Niño and La Niña, and the resultant effects of this weather on fishing activities. Each participant gave verbal consent to be interviewed.

### Meteorological data

Meteorological data were collected from the Ghana Meteorological Agency (GMet) in Tema. Climate indices of interest in this study were, sea surface temperature (SST), amount of rainfall and relative humidity. Tema meteorological data was used to run the model with the assumption that it is representative of the other landing sites. The climate data on the Tema area were from 1970 to 2015. Meteorological data were regressed with shellfish catch data obtained from FSSD of MOFAD, Tema office.

### Statistical analyses

The data collected were processed using Microsoft Excel 2010 and results presented in tables and charts. Pearson’s correlation analyses was run to show association among climate indices and shellfish catch. To develop a simple linear regression model for predicting effects of climate change on shellfish production in Ghana, a stepwise multiple regression analysis was performed on 55 years of climate and catch data using SPSS for Windows version 12.0 (SPSS, Chicago, USA).

The assumptions made in the present model were that: (i) shellfish catch is assumed to be constant and the contributions of socio-economic and other factors influencing fish catch are negligible; (ii) all important climate variables are considered; (iii) climate data on Tema area is representative of the climate of the entire coastline of Ghana.

## Results and discussion

### Historic shellfish production and climate indices

In predicting the possible effects of climate change on shellfishes, historic data on shellfish production was regressed with meteorological data of Tema.

**Figure 2.**
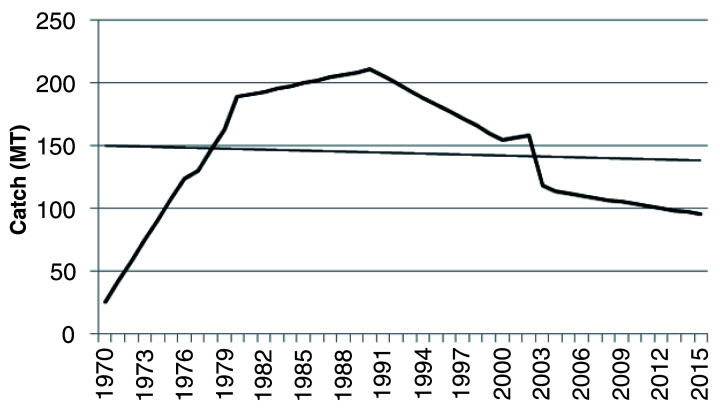
Historic shellfish catch from 1970 to 2015 in marine artisanal fisheries, Ghana.

Figure 2 illustrates trend in shellfish production and climate indices from 1970 to 2015. Production of shellfishes (excluding cephalopods) increased from 25.23 metric tonnes (Mt) in 1970 to 189.1 Mt in 1980 and 211.3 Mt in 1990, recording percentage increases in overall production of 86.66% (1970 to 1980) and 88.06% (1980 to 1990) ( Figure 2). Thereafter, there has been a 2.5-decade decline in catch by 59.65 Mt from 155.1 Mt in 2000 to 95.45 Mt in 2015. The decline in marine artisanal shell fisheries in Ghana could be due to natural and anthropogenic factors. Overfishing, climate change, low technological development, unsustainable fishing methods (such as the use of restricted fishing gears) and socio-economic factors may be reasons for this trend in production. Lack of documented data on non-commercial fish (like shellfish) and contributions from other fishery resources (oil and gas) and agricultural produce to the country's GDP could be possible reasons for the recorded reductions in shellfish catch over the years (K. Amador, personal observations).

**Figure 3.**
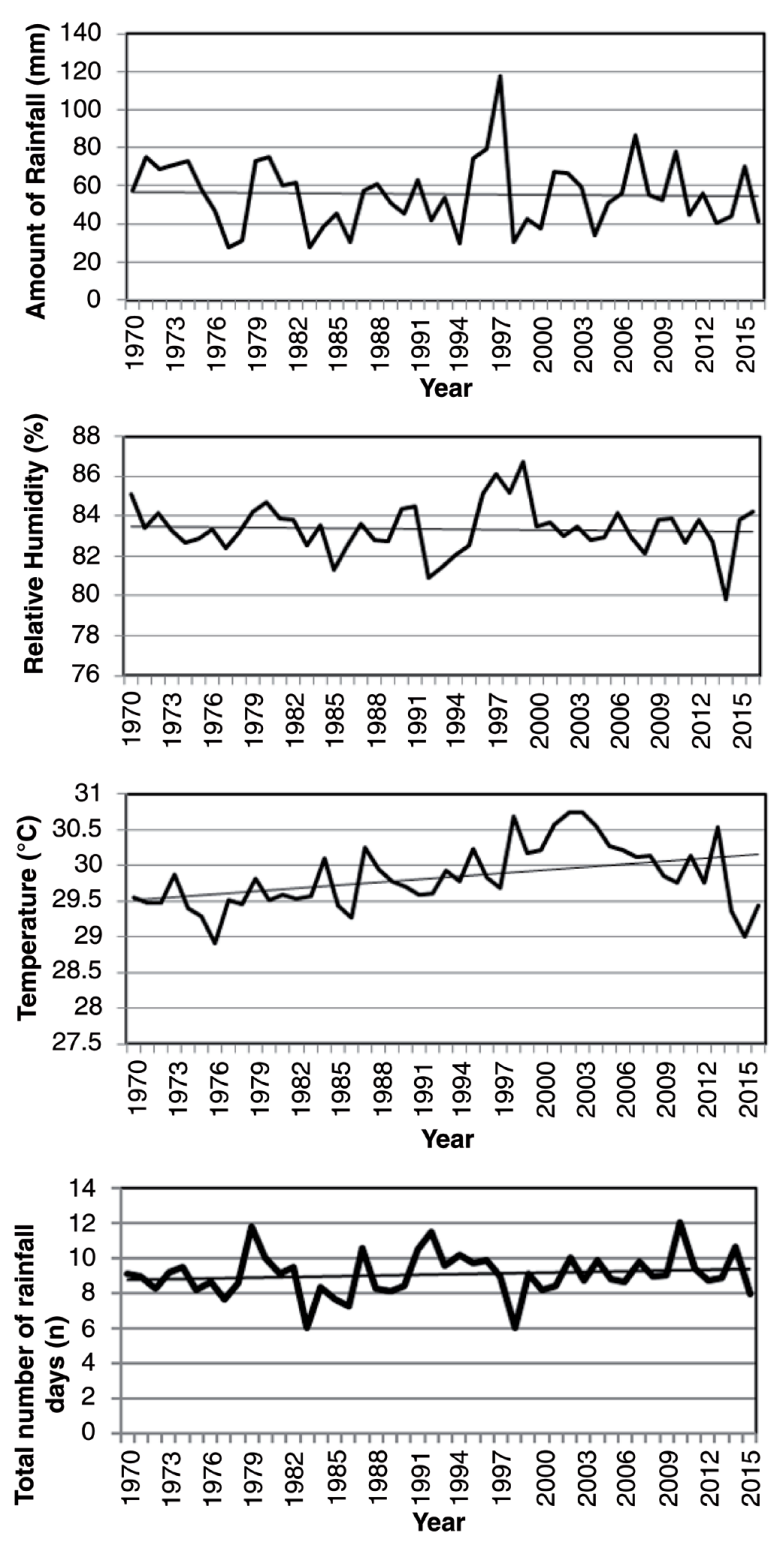
Climate indices (Tema) from 1970 to 2015 in marine artisanal fisheries, Ghana.

The amount of rainfall has reduced over the years, with values ranging between 27.45 mm and 120 mm ( Figure 3). Additionally, relative humidity has decreased in the past 55 years. It varied between 79.80% and 86.7%. In Figure 3, mean temperature shows a gradual increase and fluctuations in some years. Temperature increased from 28.90°C to 30.75°C recording an increase of about 1.85°C.

Rainfall patterns in Tema concur with the trend in rainfall for West Africa ( IDRC, 2015). The amount of rainfall has reduced over the years, with a corresponding increase in frequency of rainfall. The pattern in rainfall reflects the occurrences of El Niño (every 7–9 years) and La Niña phenomenon experienced on the coast of Ghana ( Figure 3). During these periods, there is a decline and increase in rainfall respectively. An increase in rainfall (La Niña) usually corresponds with the onset of upwelling, where nutrient-rich cold sea water is brought to the surface, resulting in a high fish catch and low temperatures, with implications on fisheries.

Relative humidity in the area also showed normal to humid conditions (79.80–86.7 %) ( Figure 3). Furthermore, mean temperature revealed increases in the recent past, with a rise in temperature of about 1.6°C. The trend in temperatures concur with the projections by the IPCC (2008). Also, studies show that Ghana will experience a discernible rise in temperature, sea level rise and concomitant decrease in rainfall in all agro-ecological zones of the country ( EPA, 2001; World Bank, 2016), similar to this study.

Variability in climate has implications on the sustainability of fisheries production, particularly temperature and rainfall. Future trends in climate may likely be a major factor influencing interrannual changes in rainfall.

### Predictive shellfish catch model

The selection approach to determine the relationship between catch and climate indices were done using the stepwise multiple linear method. The predictor variable that significantly explained shellfish catch was SST (P = 0.011) ( Table 1). The coefficient of correlation shows a positive relationship (b = 265.312) between catch and mean SST ( Table 2). This implies that as SST increases by one unit, shellfish production increase by 265.312 units. Therefore for every 1°C rise in temperature, production increases by 265 MT.

**Table 1.** Model summary relating shellfish production and mean sea surface temperature obtained from MOFAD and GMet (Tema).

Model	R	R ^2^	Adjusted R ^2^	Std. error of the estimate
1	0.956 *	0.913	0.884	29.461

*Predictors: (Constant), sea surface temperature

**Table 2.** Regression between catch and temperature obtained from MOFAD and GMet (Tema).

Model	Sum of squares	df	Mean square	Unstandardized coefficients			Standardized coefficients			
				F	Sig.	β	S.E	β	T	Sig.
Regression	27,446.153	1	27,446.153	31.622	0.011 ^b^					
Residual	2603.847	3	867.949							
Total	30,050.000	4				-				
(Constant)						7788.067			-5.534	0.012
Temperature						547.181	1407.247	0.956	5.623	0.011

df, degrees of freedom.

The multiple correlation coefficient, R, of shellfish catch against SST was 0.956 ( Table 2). The coefficient of determination, R ^2^, of the predictor variable (temperature) against catch of 0.913 indicates that 91% of the variations in shellfish catch was explained by temperature. Thus, knowing temperature, shellfish production in Ghana could be predicted. The best, most prudent simple regression model to predict catch of shellfish was described as follows:

Shellfish catch per unit effort = −7788.067 + (265.312 SST) 

The model could best be generalised from the adjusted R ^2 ^value of 0.884, which is close to the R ^2 ^value of 0.913. Therefore, if the model were derived from the population rather than a sample it would account for approximately 2.9% less variance in the outcome. Pearson correlation analyses showing the relationship between catch and rainfall, SST and humidity are shown in Table 3. There was no significant correlation between catch and amount of rainfall (P = 0.460) and catch and humidity (P = 0.393).

**Table 3.** Pearson correlation matrices showing relationship between climate indices and shellfish catch.

Statistical method	Variable	Catch	Rainfall	Temperature	Humidity
Pearson correlation	Catch	1.000	0.460	0.956	0.393
	Rainfall	0.460	1.000	0.486	0.586
	Temperature	0.956	0.486	1.000	0.168
	Humidity	0.393	0.586	0.168	1.000
Sig. (1-tailed)	Catch	-	0.218	0.006	0.256
	Rain	0.218	-	0.203	0.150
	Temperature	0.006	0.203	-	0.393
	Humidity	0.256	0.150	0.393	-
N	Catch	55	55	55	55
	Rain	55	55	55	55
	Temp	55	55	55	55
	Humidity	55	55	55	55

SST was strongly positively correlated with shellfish catch ( Table 2). The study revealed a positive correlation between shellfish catch and SST. The finding is a clear depiction of adaptation of the fish to tropical conditions in Ghana. Moderate elevations in temperature, according to Parker *et al.* (2013), may result in increase in production of some groups of shellfishes like scallops and oysters, but above optimum limits will adversely affect the growth and survival of oysters. A rise in SST affects the distribution of shellfish as a result of the weakening of materials used as attaching surfaces ( Quayle, 1989). A study by Shumway (1996) revealed that exposure of tropical oysters and other filter feeding bivalve species to temperatures above 35°C will adversely affect pumping rate and feeding. Increasing temperature affects the larval growth, immunity and fertilization of some shellfish species, while others are favoured. Temperature interferes with the absorption of carbon dioxide and has a vital role in ocean acidification. Similarly, parasitic infections are likely to result in the event of extreme elevations in temperature ( Wright *et al.*, 2011). Shellfish, particularly oysters, mussels and scallops are extremely diverse groups of organisms that are most vulnerable to adverse effects of climate change due to their inability to shift their habitats.

Tropical oysters like *Crassostrea tulipa* thrive within temperatures of 23°C and 31°C, and though little is known about the prolonged effect of temperatures above 34°C on oyster populations, from a few physiological observations, it may be inferred that continued exposure to high temperatures and high levels of carbon dioxide is unfavourable, impeding the normal rate of water transport by the gills and increasing vulnerability to diseases in higher-salinity regions ( Levinton *et al.*, 2011; Sutton *et al.*, 2012; Wright *et al.*, 2011). In linking climate scenarios to tropical gastropods such as *Littoraria* sp., the work of Chapperon & Seuront (2011) suggests that changing temperature affects the behaviour and physiology of the snail.

Considering oysters and scallops, other studies have reported similar results even within the same species, albeit at different geographical locations. For example, fertilization of Pacific oysters in Japan and Sweden were equally affected by changes in temperature and other climate factors as Australian populations of *Crassostrea gigas* despite their existence in different localities.

Conversely, other studies have revealed potential impacts from other climate determinants aside SST. For instance, a study by Meynecke *et al.* (2006) on wild-capture estuarine-dependent fish in Queensland Australia, the fish catch showed increases with corresponding increase in annual rainfall. Several authors have also reported significant positive correlations between fish catch and rainfall ( Staunton-Smith *et al.*, 2004; Vance *et al.*, 1985). Despite the observed trends, to ensure sustainable shellfisheries in Ghana, adhering to management strategies such as closed seasons, regulations on fishing methods and land use activities by resource users is pivotal in controlling harvest and the future of the industry.

Sampling efforts, seasonal changes, reproduction and other physiological changes have influences on shellfish abundance. Unfavourable conditions such as low salinity and ocean acidification leads to massive mortality among oysters and scallops ( Laakkonen, 2014). Prior studies show that relative humidity has no direct influence on shellfish abundance, but rather has indirect influences on some environmental stressors ( Levinton *et al.*, 2011; Wright *et al.*, 2011). They further reported that shellfish distribution and abundance are strongly influenced by intrinsic population characteristics such as growth rates, population densities, interactions with other organisms through competition, predation and environmental changes, which can occur simultaneously. Conclusively, a study by NOAA (2013), suggests that in the phase of distinct variability in environmental conditions, African fisheries are at risk because semi-arid countries with significant coastal and inland fisheries have high exposure to future increases in temperature.

## Conclusions

Historic shellfish production was regressed with climate indices like SST, amount of rainfall and frequency of rains to develop a model for future predictions of shell fish production in Ghana’s coastline. The model on shellfish and climate stressors explained about 91% of the variations in shellfish catch. Hence, predictive models are valuable and practical tools for understanding the dynamics of fish populations, and this predictive modelling should be considered an approach by research scientists in monitoring the effects of climate change on shellfish production along the coast of Ghana. For the sustainable management of fishery resources, forecasts are important tools in pre-empting future dynamics of an aquatic system. In ecological modelling, environmental variables are capable of shaping patterns of natural biotic populations through the creation of environmental gradients which influences the growth and survival of organisms ( Rocha *et al.*, 2009). This study showed that there was no correlation between relative humidity or amount of rainfall and shellfish production. However, these factors, together with other environmental stressors, also affect production and as such should not be disregarded in shell fish stock management. Additionally, to our knowledge this is the first model developed for shellfish in the country to consider a number of assumptions. It is therefore recommended that this model be revised with time for accuracy and precision. 

Agencies and Ministries responsible for fisheries and water use governance and sustenance in Ghana, such as the Ministry of Fisheries and Aquaculture Development, Wildlife and Forest Division and Water Resources Commission should promote and utilize the predictive model developed in this study in the management of coastal fisheries resources.

The sensitivity of shellfish harvesters to climate variability, its impact on shell fish abundance and the existing strategies necessary to facilitate the uptake of coping and adaptive strategies is essential to minimize adverse impacts of climate change. In future, we will be looking at the use of oysters (West African oyster) as a bio indicator of environmental variability in estuaries in Ghana. We can decipher the need to investigate socioeconomic issues influencing fish abundance and adaptation strategies among fishers for a sustainable shell fish production in Ghana under our changing climate.

## Data Availability

Open Science Framework: Modelling the effects of climate change on shellfish production in marine artisanal fisheries of Ghana. https://doi.org/10.17605/OSF.IO/SHDK2 ( Atindana, 2019). This project contains raw data on mollusc catch and climate assessed in this study. Data are available under the terms of the Creative Commons Zero "No rights reserved" data waiver (CC0 1.0 Public domain dedication).
