# Supplementary material for: Modelling the effects of climate change on shellfish production in marine artisanal fisheries of Ghana
Source: AAS Open Res. 2025 Jan 16;2:16. Originally published 2019 May 22. [Version 2] doi: 10.12688/aasopenres.12956.2 (PMC12038342; doi:10.12688/aasopenres.12956.2)
Supplement: Peer review history

# Reviewer report: Reviewer response for version 2

**DOI:** 10.21956/aasopenres.16841.r32160

**Published:** 2025-04-28

**Authors:** Ruby Asmah

**Affiliations:** 1 Water Research Institute, Council for Scientific and Industrial Research, Accra, Greater Accra Region, Ghana

The manuscript sought to model the impact of climate change on shellfish production from capture, along the coast of Ghana. Shellfish yearly production data from 1970 to 2015 was used. Missing annual production figures were estimated based on proportionality of total fish catch to shellfish.  Although four sites were selected for evaluation, the climate models were simulated using climate data from just one of the sites.

 The climate-production regression model to predict the impact of climate were based on annual averages. Climate predictions are more accurate with long term high frequency data. I will propose that the author use at least monthly climate and shellfish production data to run the models. Predictions from such models would be near accurate.

Is the work clearly and accurately presented and does it cite the current literature?

Partly

If applicable, is the statistical analysis and its interpretation appropriate?

I cannot comment. A qualified statistician is required.

Are all the source data underlying the results available to ensure full reproducibility?

Yes

Is the study design appropriate and is the work technically sound?

Partly

Are the conclusions drawn adequately supported by the results?

Partly

Are sufficient details of methods and analysis provided to allow replication by others?

Yes

Reviewer Expertise:

Aquaculture and the environment

I confirm that I have read this submission and believe that I have an appropriate level of expertise to confirm that it is of an acceptable scientific standard, however I have significant reservations, as outlined above.

# Reviewer report: Reviewer response for version 1

**DOI:** 10.21956/aasopenres.14034.r27325

**Published:** 2020-01-31

**Authors:** Doris Soto

**Affiliations:** 1 Interdisciplinary Center for Aquaculture Research (INCAR), University of Concepción, Concepción, Chile

**General comments:**

- The statistical analysis seems well done and the information used is appropriate and also the general conclusion that there is a correlation between SST and catch is adequate but there is not enough information to support causality. That is to support that SST or climate change can determine catch.
- The main concern of this report is that it is not clear; it is not known how much of the fishery decline is due to overfishing. It is quite possible that as SST increased there was also an increase in fishing pressure. It would be necessary to have at least some description of the number of fishermen or some information on fishing efforts during the years to disentangle the potential joint effects of climatic variability, climatic trend and overfishing.
- For example, increasing SST may affect other resources (fishery resources or land resources, agriculture) and thus people can go more often to the sea searching for food and alternative livelihoods.
- If the fishing pressure can be assumed to be constant through the years then there is no problem but this information is needed or at least some discussion is necessary. 
- In page 7 there is a formula referring to catch per unit effort but it is not clear how the authors get there. The authors must have that information on fishing effort. Perhaps is a matter of clarification but this is important especially to further designing of relevant policies to increase fishery resilience.

Is the work clearly and accurately presented and does it cite the current literature?

Yes

If applicable, is the statistical analysis and its interpretation appropriate?

Partly

Are all the source data underlying the results available to ensure full reproducibility?

Partly

Is the study design appropriate and is the work technically sound?

Partly

Are the conclusions drawn adequately supported by the results?

Partly

Are sufficient details of methods and analysis provided to allow replication by others?

Partly

Reviewer Expertise:

Climate change impacts on fisheries and aquaculture climate change adaptation, vulnerability analysis, ecosystem approach to fisheries and aquaculture

I confirm that I have read this submission and believe that I have an appropriate level of expertise to confirm that it is of an acceptable scientific standard, however I have significant reservations, as outlined above.

# Response

**Published:** 2020-06-16

**Authors:** SANDRA AKUGPOKA ATINDANA

**Affiliations:** C K Tedam University of Technology and Applied Sciences, Navrongo, Upper East Region, Ghana

Dear reviewer,

As part of the set assumptions for the model, assumption 1, explains capture factors such as overfishing and its effect as held constant. That is the reason why there was no discussion on fishing pressure. As part of our future aim, under the conclusion section of this manuscript, our upcoming research will address the effects of overfishing and other socio-economic factors such as fishing efforts on oyster fisheries in Ghana.

# Reviewer report: Reviewer response for version 1

**DOI:** 10.21956/aasopenres.14034.r27167

**Published:** 2019-09-05

**Authors:** Kobina Yankson

**Affiliations:** 1 University Of Cape Coast, Cape Coast, Ghana

The article is recommended for publication. Desktop data was largely used and the sources indicated are reliable. However, regarding the availability of the data sources for full reproducibility, I indicated "partly" because all molluscs were lumped together. Since individual species differ in their responses to environmental changes, it is not likely to reproduce the exact results always.

- Appropriate statistical analyses were employed to analyse the results.
- The conclusions drawn are sound and supported by the results.
- I however, noted some typographical errors which I am not sure where to deal with them (see below).
- Also, in the paragraph directly under Figure 3, the last sentence needs a slight correction.  Actually, the onset of upwelling is after the rainy season and does not correspond with it as stated.

Some typographical corrections:

 Under Introduction:

- 2 ^nd^ paragraph, line 10: Change “staunched” to “staunch”.

Under Study Area:

- 1 ^st^ line: Delete “region” after Volta.
- The location of Elmina, one of the sampling sites, has not been described as done for the three other sites. It should be described.

Under Results and discussion:

- 2 ^nd^ paragraph, 3 ^rd^ line from bottom: Change “other fishery resources” to “other non-fishery resources”. (Note that oil and gas are non-fishery).
- 1 ^st^ paragraph under Predictive Shellfish Catch Model: Line 1: Change “were done” to “was done”.

“Shellfish” should be written as one word throughout the text; not “Shell fish”.

Is the work clearly and accurately presented and does it cite the current literature?

Yes

If applicable, is the statistical analysis and its interpretation appropriate?

Yes

Are all the source data underlying the results available to ensure full reproducibility?

Partly

Is the study design appropriate and is the work technically sound?

Yes

Are the conclusions drawn adequately supported by the results?

Yes

Are sufficient details of methods and analysis provided to allow replication by others?

Yes

Reviewer Expertise:

Marine Ecology, Bivalve biology and oyster culture

I confirm that I have read this submission and believe that I have an appropriate level of expertise to confirm that it is of an acceptable scientific standard.
